# Clustering analyses in peptidomics revealed that peptide profiles of infant formulae are descriptive

**DOI:** 10.1002/fsn3.196

**Published:** 2014-12-30

**Authors:** Tim T Lambers, Jolein Gloerich, Els van Hoffen, Wynand Alkema, Dirk H Hondmann, Eric AF van Tol

**Affiliations:** 1 Mead Johnson Pediatric Nutrition InstituteNijmegen, The Netherlands; 2 Radboud Proteomics Center, Department of Laboratory Medicine, Radboud University Medical CenterNijmegen, The Netherlands; 3 NIZO food research B.V.Ede, The Netherlands

**Keywords:** Bioactive peptides, infant nutrition, peptidomics, protein hydrolysates

## Abstract

Prompted by the accumulating evidence on bioactive moieties of milk-derived peptides, novel methods were applied to compare the peptide composition among commercially available hydrolysate formulations and to determine batch-to-batch variations of protein hydrolysate products. Despite the availability of general methods to measure, for example, the degree of hydrolysis and peptide mass distribution at a high level, the objective of this study was to more qualitatively compare peptide sequences and composition. By a comprehensive approach combining peptidomics technologies and multivariate clustering analyses, the peptide profiles of different hydrolyzed milk protein formulations were compared. Moreover, peptide profiles of various hydrolysate batches that had been produced over a period of 5 years were included. Coupling of identified peptide sequences to the position in their corresponding milk proteins produced numerical datasets that subsequently were utilized for multivariate data analyses. These analyses revealed that batch-to-batch variation in the peptide profiles of a specific extensively hydrolyzed casein preparation was low. Moreover, extensive multivariate evaluations revealed that the peptide profiles of different commercially available hydrolyzed milk protein formulations provided a descriptive and distinct signature. Overall, the described methodology may contribute to the field of peptide research as observed dissimilarities in peptide profiles of similar products may be related to differences in their overall functionality.

## Introduction

Peptides occur widely in nature and play a significant role in numerous biological functions, for example, as hormones or signaling molecules. Human and bovine milk peptides can elicit beneficial physiological effects which extend beyond their nutritional value (Baldi et al. 2005; Rutherfurd-Markwick 2012). These peptides can be naturally present in human or bovine milk, formed during gastrointestinal digestion, or generated through processes of protein hydrolysis for specific nutritional applications (Roncada et al. 2012; Dallas et al. 2013; Wan et al. 2013). A diverse range of physiological benefits have been assigned to such milk-derived peptides, including effects on the digestive, immune, and nervous system (Wada and Lonnerdal 2014; Raikos and Dassios 2014). Within the infant food category, many different hydrolysate formulations are available for, for example, the dietary management of cow's milk allergy. Novel insights into the hydrolysate peptide composition as well as functionality will further contribute to the understanding of their biological activity and potential role in reducing allergic manifestations and accelerating tolerance acquisition to cow's milk proteins (Berni Canani et al. 2012; von Berg et al. 2013).

Currently, protein hydrolysates are mainly classified by their protein source and degree of hydrolysis. Herewith, the terms partial and extensive are commonly used to classify whether the degree of hydrolysis is generally low or high, respectively. Additional nonclinical characteristics are typically provided by chromatographic mass distribution analyses that provide a general, high level, overview of the peptide mass distribution (Leary 1992). The distinctive capacity of such analytical technologies, however, is rather poor, and generally, mass distributions are described at kDa level. More sensitive technologies based upon automated Edman degradation are applied to determine peptide-length distribution profiles, providing a more detailed description (Siemensma et al. 1993). Although sensitive and comprehensive, these methods do not deliver peptide sequence information. More detailed characterization based upon peptide sequences may, however, be warranted given the increasing understanding of the relevance of specific sequences in these hydrolysates for overall biological activity.

Peptidomics and the identification of peptides have become a technology that has found its application in many research areas due to the rapid development of mass spectrometry-based tools and methodologies. It excels as one of the most informative methods for peptidome analyses as it enables identification of multiple peptides simultaneously with high sensitivity even in complex matrixes such as food or biological fluids (Schrader and Schulz-Knappe 2001; Baggerman et al. 2004; Ivanov and Yatskin 2005). The large datasets produced from an individual measurement, however, may pose significant challenges in extracting specific information and the overall comparison of different datasets remains challenging. In this respect, a number of bioinformatics methods that are able to handle multiple dimensional data arising from OMICS platforms can be adopted (Chadeau-Hyam et al. 2013) and recently combinations of peptidomics and bioinformatics have been developed (Norden et al. 2005; Schmidt et al. 2008; Menschaert et al. 2009).

In this study, combinations of peptidomics and multivariate clustering analyses were applied to compare peptide profiles of milk protein hydrolysates and formulae thereof. Although enzymatic hydrolysis of cow's milk proteins at industrial scale is well-controlled, and the degree of hydrolysis is quite reproducible, little is known about the specific peptide composition. This study demonstrates that, with respect to peptide composition, the batch-to-batch variation of a specific extensively hydrolyzed casein (eHC) sample produced over a 5-year period is low. Moreover, extensive profiling of several commercially available infant formulae for the dietary management of cow's milk allergy reveals that their peptide profiles provide a descriptive and distinct signature. Overall, the methodologies described in this study may find application in (food science or quality control) peptidomics in order to compare peptide profiles, as observed dissimilarities in peptide profiles may, for example, be related to differences in overall biological functionality.

## Materials and Methods

### NanoLC-MS/MS analyses

Peptide profiles were generated by nanoLC-MS/MS analyses of four batches of several commercially available eHC formulae (1: Nutramigen, Mead Johnson Nutrition; 2: Allergycare, FRISO and 3: Similac Alimentum, Abbott; obtained from a local pharmacy), an extensively hydrolyzed whey (eHW) formula (Nutrilon Pepti, Nutricia) and different production batches of a specific extensively hydrolyzed casein (Nutramigen hydrolysate; Mead Johnson Nutrition) sampled over a 5-year period. To ensure that the formulae contained hydrolysates from different production batches, formulae were obtained that had at least a 3-month different “best before” date. eHC batches were sampled over a 5 year period: September 2007, April 2008, September 2009, July 2010, and April 2011.

Samples were suspended in 0.1% formic acid to a concentration of 1 *μ*g/*μ*L by sonication in a water bath for 10 min. Reduction of possible cysteine disulfide bridges was performed by adding 2 *μ*L 10 mmol/L dithiothreitol to a total of 5 *μ*L hydrolysate solution (5 *μ*g) and incubating for 30 min at room temperature. Alkylation of reduced cysteine residues was performed by adding 2 *μ*L 50 mmol/L chloroacetamide and incubating the samples for 20 min in the dark. The resulting peptide mixtures were desalted and concentrated using stop and go elution (STAGE) tips according to Rappsilber et al. (2003). Finally, samples were resuspended in 20 *μ*L 0.1% formic acid prior to nanoLC-MS/MS measurements.

NanoLC-MS/MS analyses were performed using an EASY-nLC liquid chromatograph (Thermo Fisher Scientific Inc., Waltham, MA USA), coupled online via a nanoelectrospray ion source (Thermo Fisher Scientific Inc., Waltham, MA USA) to a 7T linear ion trap Fourier transform ion cyclotron resonance mass spectrometer (LTQ FT Ultra; Thermo Fisher Scientific). Instrument settings are further explained in Data S1. Mass spectrometric data files were searched using the database search program Mascot (version 2.2; Matrix Science Inc., London, UK). The database used for the searches consisted of a consensus bovine milk protein database as described by D'alessandro et al. (2011), with addition of known contaminants such as human keratins. As input for bioinformatics analyses, summed intensities over the measured m/z range were extracted across the chromatographic gradient (m/z-summed intensity). Alternatively, database search peptide identifications derived from the major milk proteins *α*S1-casein (P02662), *α*S2-casein (P02663), *β*-casein (P02666), *κ*-casein (P02668), *α*-lactalbumin (P00711), and *β*-lactoglobulin (P02754) with an ions score >20 were considered significant and included for bioinformatics analyses.

### Peptide-length distribution

Peptide lengths and their relative abundance were determined by automated Edman degradation and amino acid determination as described previously (Siemensma et al. 1993).

### Bioinformatics

Peptide sequences were mapped to their corresponding position in the proteins from the database to produce numerical datasets for bioinformatics analyses. Peptide coverage was calculated for each amino acid in the database for subsequent statistical analyses and clustering. Statistics, principal component analyses (PCA), and hierarchical clustering were performed with the statistical software package R (http://www.r-project.org). PCA were performed on both normalized and nonnormalized datasets. Normalization of the datasets was done per sample by *Z*-transformation.

## Results and Discussion

**Figure 1:**
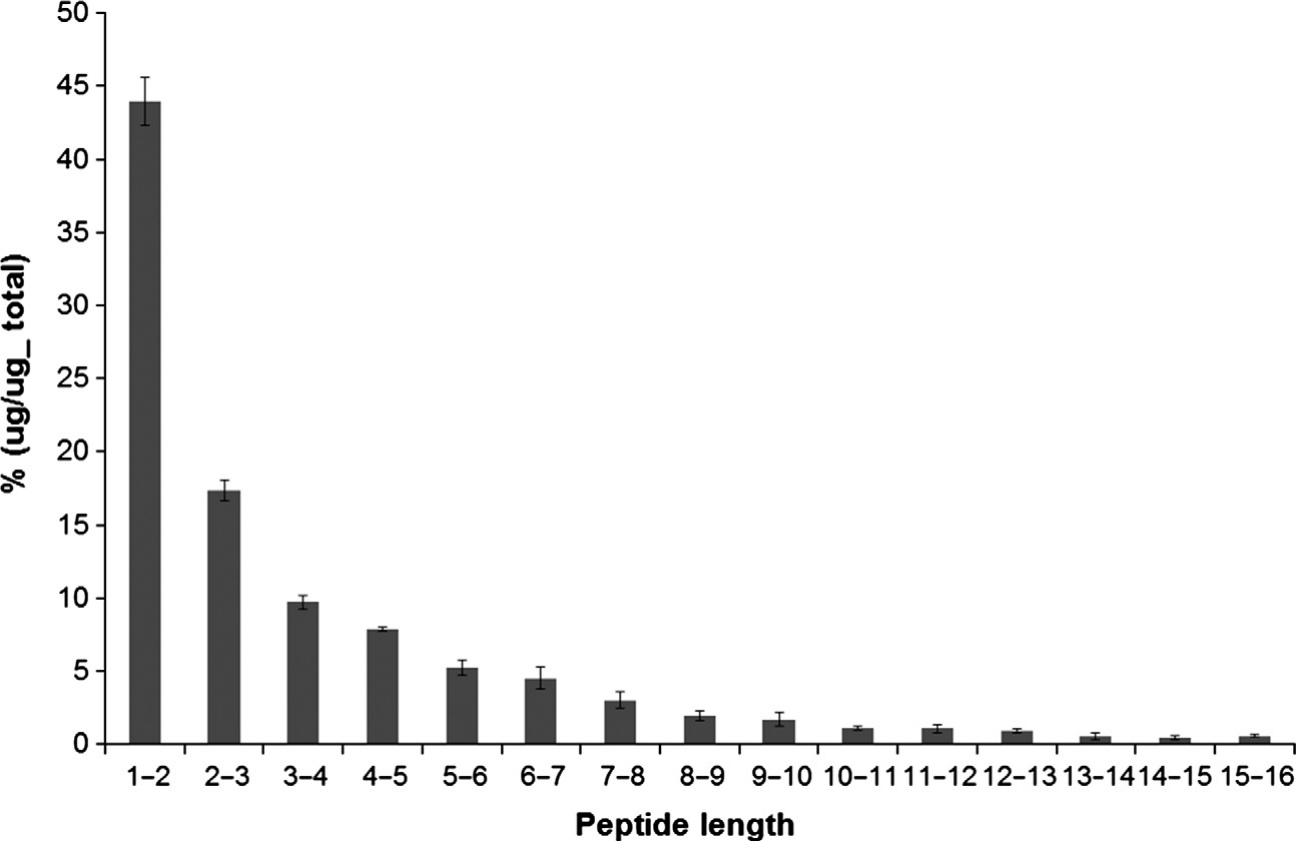
Peptide-length distribution of extensively hydrolyzed casein formula 1. Peptide lengths of extensively hydrolyzed casein formula 1 (*n* = 4) presented as *μ*g percentage of total.

Hydrolysate formulae are mainly characterized by their protein source and degree of hydrolysis. Prompted by the accumulating evidence on bioactive moieties of milk-derived peptides, novel methods were explored to compare the peptide composition among commercially available hydrolysate formulations. Of particular interest was the group of formulae applied for the dietary management of cow's milk allergy. Many of these formulae contain extensively hydrolyzed protein sources to decrease overall allergenicity (Dupont et al. 2012; Ludman et al. 2013). Typically such formulae contain a large proportion of smaller sequences as illustrated by the peptide-length distribution (Fig.** **1). Nonetheless, longer peptide sequences can be identified that, from an immune modulatory perspective, may be of interest given their possible capacity to bind MHC molecules (Felix and Allen 2007). Peptide profiles from three commercially available eHC infant formulae (*n* = 4 of each formula brand) were determined by nanoLC-MS/MS analyses. As a control one eHW formula (*n* = 4) was analyzed that based upon the source (whey rather than casein) should provide a different peptide signature. Overall coverage of the peptide identifications, that is, the number of peptide identifications compared to the observed number of ions, was relatively low compared to standard tryptic protein digests. This can be explained by the fact that extensively hydrolyzed protein sources (generated with unknown or combinations of different proteases) generally contain a higher number of smaller peptide sequences without terminal charged amino acids, as compared to tryptic digests. Together, these peptides are often detected as 1+ ions in LC-MS analyses. Further fragmentation of 1+ spectra during LC-MS/MS analyses result in lower quality spectra than for 2+ ions, which can hamper identification of the peptide. Furthermore, smaller peptides generate fewer fragments in MS/MS spectra and thus database search score of these peptides is intrinsically lower than for longer sequences.

**Figure 2:**
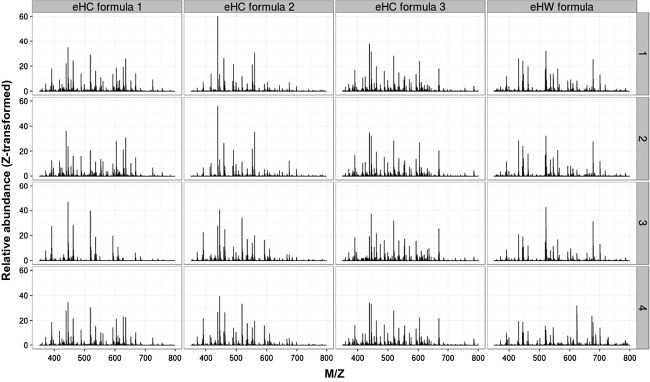
LC-MS profiles of extensively hydrolyzed casein and extensively hydrolyzed whey formulae. LC-MS profiles of all individual extensively hydrolyzed casein and extensively hydrolyzed whey formulae (*n* = 4 of each formula). Summed intensities over the measured m/z range were extracted across the chromatographic gradient and datasets were normalized by *Z*-transformation.

**Figure 3:**
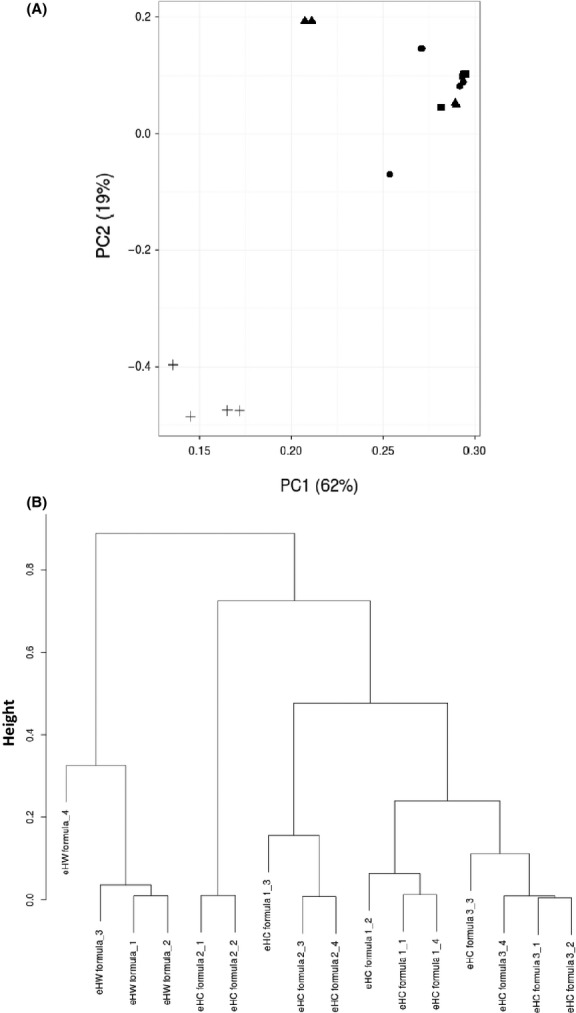
Multivariate clustering analyses of LC-MS profiles from extensively hydrolyzed casein and extensively hydrolyzed whey formulae. Principal component analysis (A) and hierarchical clustering of the corresponding similarity–dissimilarity matrix (B) from normalized (*Z*-transformed) LC-MS profiles (summed intensities over the measured m/z range) of extensively hydrolyzed casein and extensively hydrolyzed whey formulae; eHC formula 1 (●); eHC formula 2 (▲); eHC formula 3 (■) and eHW formula (+).

Initially, LC-MS data from the measurements were subjected to multivariate clustering analyses, as this dataset already illustrated differences in-between the formulae (Fig.** **2). Whereas LC-MS profiles of eHC formula 1 and eHC formula 3 seem most comparable, those of eHC formula 2 and eHW formula appear different from the others. Although biased by irrelevant data points from ions that originate from nonproteinaceous material, minor milk proteins normally lost in database identifications or known contaminants including keratins, comparing LC-MS datasets allows a close to full spectrum analysis given the loss of coverage with database-driven peptide profiling. PCA plots of LC-MS datasets revealed that clusters of the eHW-based formula and eHC formulae can be observed (Fig.3A). The first and second principal components explained the majority (81%) of the variation and further differentiation of the different formulae was not observed with other components (data not shown). To establish a more quantitative measure of similarities and dissimilarities and to investigate if differences occurred within the casein group as suggested from the LC-MS profiles, all pairwise correlations between the individual LC-MS datasets were calculated. Hierarchical clustering of this correlation matrix suggests, at least to some extent, that further differences between the individual samples within the casein formulae group exist (Fig.3B). Two main clusters were distinguished consisting of the eHW formula and eHC formulae. Secondly, other formula types all containing eHC were found to be clustered with formulae 1 and 3 being the most similar. In this particular analysis, complete differentiation between eHC formula 1 and eHC formula 2 as suggested by the LC-MS profiles appeared not possible.

**Figure 4:**
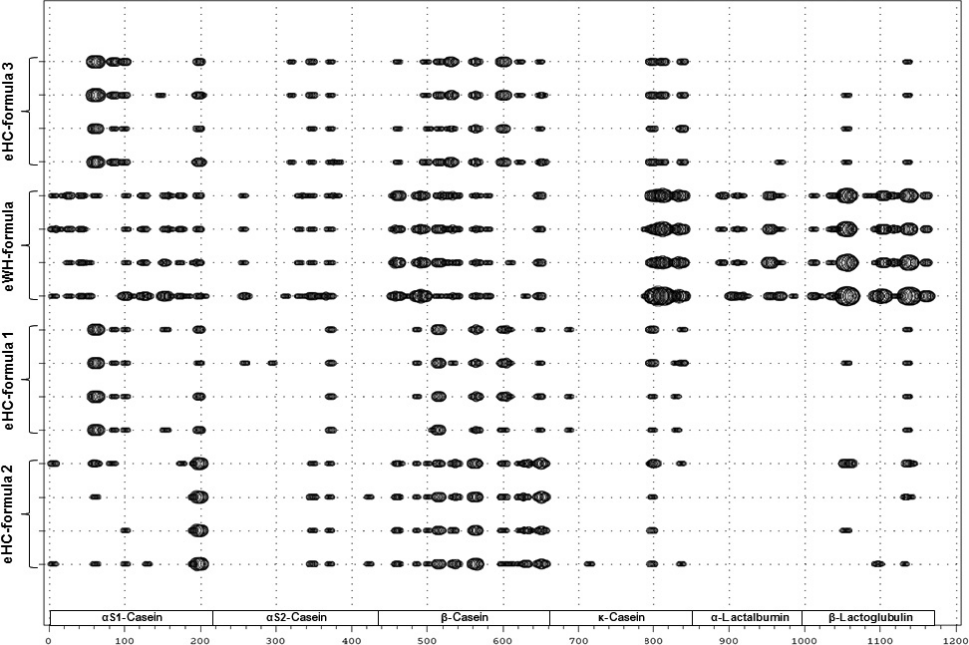
Peptide coverage in major milk proteins. Peptide profiles of different infant formulae were determined and peptide coverage for peptides with ions score >20 were calculated for each amino acid of the major milk proteins *α*S1-casein (P02662), *α*S2-casein (P02663), *β*-casein (P02666), *κ*-casein (P02668), *α*-lactalbumin (P00711), and *β*-lactoglobulin (P02754) as depicted in bubble plots of the consecutive protein sequences. The size of the bubble represents the count at the corresponding position in the protein.

**Figure 5:**
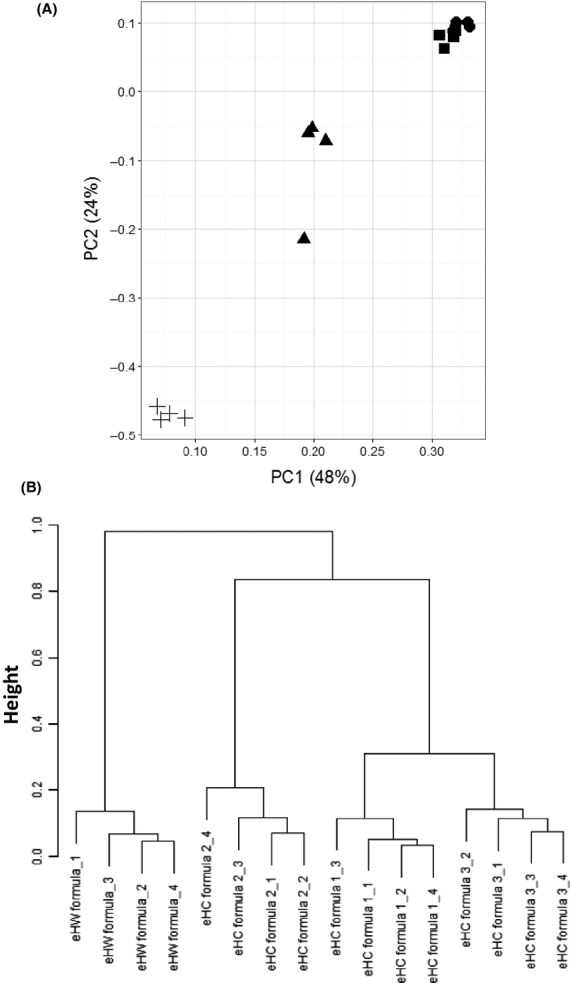
Principal component analyses of formulae peptide profiles. Peptide profiles were determined and peptide coverage was calculated for each amino acid of the major milk *α*S1-casein (P02662), *α*S2-casein (P02663), *β*-casein (P02666), *κ*-casein (P02668), *α*-lactalbumin (P00711), and *β*-lactoglobulin (P02754). Principal component analyses were performed for datasets with an ions score >20 (A) and hierarchical clustering of the corresponding similarity–dissimilarity matrix (B) were determined. eHC formula 1 (●); eHC formula 2 (▲); eHC formula 3 (■) and eHW formula (+).

Given the possible overlap at the LC-MS level in the eHC formula group and the absence of a clear differentiation, peptide identification profiles (from LC-MS/MS datasets) were further explored. Identified peptide sequences were mapped to their position in the corresponding major milk protein sequence from the database to create numerical datasets for statistical comparison (Fig.** **4). Although based upon casein, whey-derived sequences were detected in the peptide profiles of eHC formulae. Vice-versa, casein-derived sequences were detected in eHW-based formulae. These observations can be explained by the fact that many industrial casein sources contain small amounts of whey proteins and vice-versa that can eventually be discriminated in hydrolysates thereof by proteomics technologies. PCA plots revealed that peptide profiles from the major milk proteins of all formulae types can be distinguished as individual clusters with the peptide profile of the eHW-based formula being the most isolated (Fig.** **5A). Strikingly, all eHC formulae, although relatively closer than the eHW formula, are recognized as individual clusters suggesting that the profiles provided a descriptive signature. The profiles of eHC formulae 1 and 3 appeared most related, which fits with the clustering at LC-MS level. To establish a more quantitative measure of the similarities and dissimilarities, all pairwise correlations between the individual samples were calculated using the mapping data. Hierarchical clustering of these correlation matrixes confirmed that peptide profiles of all tested formula types provide a descriptive signature (Fig.** **5B). These trees graphically highlight the clusters of the individual samples of the products. At high level, two clusters are discriminated consisting of the eHW formula and eHC formula. Furthermore, individual formula types containing eHC are recognized as separate clusters with formulae 1 and 3 being the most similar. Similar clustering results were obtained when peptide identification thresholds were lowered (i.e., ions score >10; data not shown) suggesting that, although likely resulting in inclusion of false-positive identifications, identification threshold in peptidomics may be lowered for overall comparison of datasets. The latter may possibly be warranted when overall quality of the peptidomics spectra (e.g., when dealing with challenging samples such as extensively hydrolyzed protein sources, complex matrixes etc.) is low and ions scores with the identifications are reduced.

Overall, adding to the available nonclinical testing methodologies, the described combination of peptidomics and multivariate clustering analyses thus provides a method to compare infant formula at the peptide sequence levels not possible with established methodologies such as mass distribution analyses and Edman degradation-based peptide-length distribution (Leary 1992; Siemensma et al. 1993).

**Figure 6:**
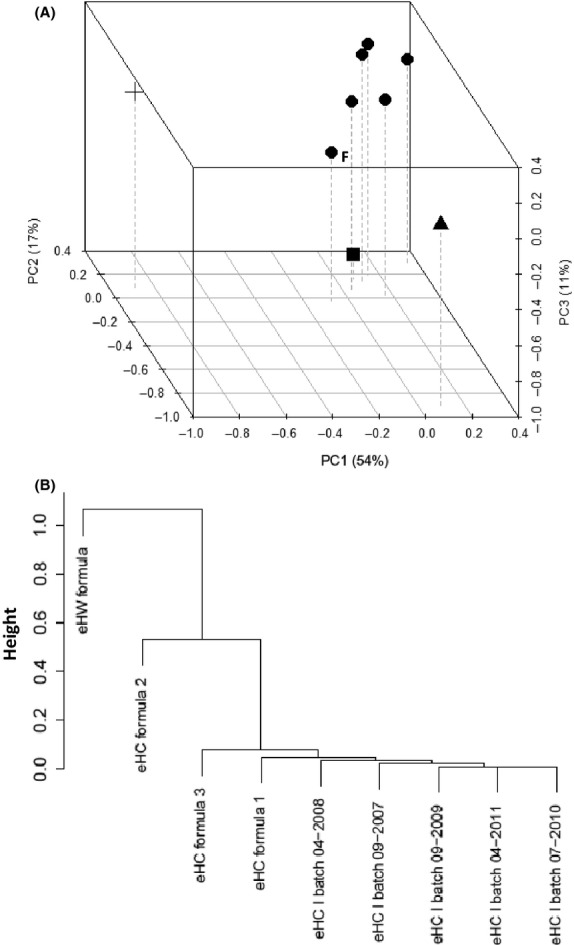
Multivariate clustering analyses of peptide profiles from different hydrolysate production batches. Peptide profiles of several infant formulae and different production batches of a specific extensively hydrolyzed casein as sampled over a 5-year production period were determined and peptide coverage was calculated for each amino acid of the major milk *α*S1-casein (P02662), *α*S2-casein (P02663), *β*-casein (P02666), *κ*-casein (P02668), *α*-lactalbumin (P00711), and *β*-lactoglobulin (P02754). Principal components analyses were performed for datasets with an ions score >20 (A) and hierarchical clustering of the corresponding similarity–dissimilarity matrix (B) were determined. eHC hydrolysate batches (●); eHC formula 1 (F); eHC formula 2 (▲); eHC formula 3 (■) and eHW formula (+).

To further test application of these descriptive peptidomics methods in infant food manufacturing, we next explored batch-to-batch variation within a hydrolysate production process. Peptide profiles of infant formulae likely contribute to overall formula functionality and hence large variations in these profiles may be unwanted. Industrial preparation of protein hydrolysates is, however, a delicate process with possible natural variations in milk protein sources, protease activities, etc. Given these possible variations it is therefore likely that certain batch-to-batch differences may occur which may become apparent when applying extremely sensitive analyses such as peptidomics. To gain insight in batch-to-batch variations, we sampled a specific eHC hydrolysate during a 5-year period and applied the above-described methods to compare peptide profiles in relation to the peptide profile of several hydrolysate formulae. By mapping identified peptide sequences to their position in the corresponding protein sequence from the database, numerical datasets were produced as input for multivariate clustering analyses (Fig. ** **6). Principal component analysis was applied to study overall clustering of peptide profiles from the individual hydrolysate batches. As compared to the profiles of several formulae, the peptide profiles of the different batches reveal a distinctive cluster together with eHC formula 1, the formula containing this particular hydrolysate. In contrast, peptide profiles of eHC formulae 2 and 3 are more distant (most evident in the first and third principal component). As expected, the profile of the whey hydrolysate-based formula is most distant. To establish a more quantitative measure of the similarities and dissimilarities, all pairwise correlations between the individual samples were calculated. Hierarchical clustering of these correlations further confirmed that peptide profiles of different hydrolysate batches are highly similar, as well as to finished eHC formulation 1 that contains this particular casein hydrolysate. Furthermore, correlation of the hydrolysate batches with the other eHC formulae is lower and virtually absent with the eHW formula.

## Conclusion

The current work describes that a combination of mass spectrometry-based peptidomics and multivariate clustering analyses allows for a comprehensive comparison of hydrolyzed milk protein formulae at the peptide level. Whereas current comparative compositional analyses are mainly restricted to chromatographic mass distribution analyses or Edman degradation-based peptide-length measurements, the described methodology allows comparing hydrolyzed milk protein formulae at the peptide sequence level. Formula peptide profiles were found to provide a descriptive and distinct signature. Furthermore, with respect to peptide composition, the batch-to-batch variation of a specific eHC preparation produced over a 5-year period was low. Overall, the descriptive methodology may contribute to the field of peptide/hydrolysate research as observed dissimilarities in peptide profiles of products may relate to differences in overall functionality. Additionally, applications may be found in quality control to gain insight in batch-to-batch variation and effects of different steps in the production process on the overall peptide profile.

## Supporting Information

Data S1 Supplemental experimental procedures.
