# Supplementary material for: Clustering analyses in peptidomics revealed that peptide profiles of infant formulae are descriptive
Source: Food Sci Nutr. 2014 Dec 30;3(1):81–90. doi: 10.1002/fsn3.196 (PMC4304566; doi:10.1002/fsn3.196)
Supplement: Data S1 — Supplemental experimental procedures. [file fsn30003-0081-sd1.docx]

**Supplemental experimental procedures**

This table lists the nanoLC-MS/MS measurement configuration and settings:

| **nanoLC settings** |  |
| --- | --- |
| Column | Trap column: Acclaim PepMap100, 5 μm, 100 Å, 300 μm i.d. × 5 mm |
|  | Analytical column: 15 cm fused silica emitter (New Objective, Tip: 8 +/- 1 μm, ID: 100 μm) packed with 3 μm Reprosil C18 beads |
| LC method | Gradient (total 76 minutes): |
|  | - From 0% buffer B to 5% buffer B in 1 minute  - From 5% buffer B to 35% buffer B in 60 minutes  - From 35% buffer B to 80% buffer B in 5 minutes |
|  | Column washing:  - 80% buffer B for 10 minutes |
| Buffer A | 0.1% formic acid |
| Buffer B | 0.1% formic acid in acetonitrile |
| Flow rate | 300 nl/min |
| **MS settings** |  |
| Data-dependent mode | Sequencing of the four most abundant ions |
| Dynamic exclusion | 360 s, 1 repeat count, 1.5 amu precursor mass tolerance |
| Mass range FT ICR MS | 350-1,600 m/z |
| Resolution FT ICR MS | 100.000 |
| Charge state rejection | 4+ and unassigned charge states |
| **Database search settings (Mascot)** |  |
| Enzymatic cleavage | None |
| Precursor ion tolerance | 10 ppm |
| Fragment ion tolerance | 0.5 Da |
| #^13^C | 1 |
| Charge state | 1+, 2+ and 3+ |
| Fixed modification | Carbamidomethylation (C) |
| Variable modifications | Oxidation (M), Acetyl (Protein N-term), Phosphorylation (STY) |
